# Nano-Tailored Triple Gas Sensor for Real-Time Monitoring of Dough Preparation in Kitchen Machines

**DOI:** 10.3390/s25092951

**Published:** 2025-05-07

**Authors:** Dario Genzardi, Immacolata Caruso, Elisabetta Poeta, Veronica Sberveglieri, Estefanía Núñez Carmona

**Affiliations:** 1 Institute of Bioscience and Bioresources (CNR-IBBR), National Research Council, Via J.F. Kennedy, 17/i, 42124 Reggio Emilia, RE, Italy; dario.genzardi@ibbr.cnr.it (D.G.); estefania.nunezcarmona@cnr.it (E.N.C.); 2 Department of Engineering “Enzo Ferrari”, University of Modena and Reggio Emilia, Via Pietro Vivarelli, 10, 41125 Modena, MO, Italy; 3 Institute of Bioscience and Bioresources (CNR-IBBR), National Research Council, Via Madonna del Piano, 10, 50019 Sesto Fiorentino, FI, Italy; 4 Department of Life Sciences, University of Modena and Reggio Emilia, Via J.F. Kennedy, 17/i, 42124 Reggio Emilia, RE, Italy; elisabetta.poeta@unimore.it; 5 Nano Sensor Systems s.r.l. (NASYS), Via Alfonso Catalani 9, 42124 Reggio Emilia, RE, Italy

**Keywords:** leavening, fermentation, IoT system, volatile organic compounds (VOCs) analysis, MOS gas sensors

## Abstract

We evaluated the efficacy of an innovative technique using an S3+ device equipped with two custom-made nanosensors (e-nose). These sensors are integrated into kitchen appliances, such as planetary mixers, to monitor and assess dough leavening from preparation to the fully risen stage. Since monitoring in domestic appliances is often subjective and non-reproducible, this approach aims to ensure safe, high-quality, and consistent results for consumers. Two sensor chips, each with three metal oxide semiconductor (MOS) elements, were used to assess doughs prepared with flours of varying strengths (W200, W250, W390). Analyses were conducted continuously (from the end of mixing to 1.5 h of leavening) and in two distinct phases: pre-leavening (PRE) and post-leavening (POST). The technique was validated through solid-phase micro-extraction combined with gas chromatography–mass spectrometry (SPME-GC-MS), used to analyze volatile profiles in both phases. The S3+ device clearly discriminated between PRE and POST samples in 3D Linear Discriminant Analysis (LDA) plots, while 2D LDA confirmed flour-type discrimination during continuous leavening. These findings were supported by SPME-GC-MS results, highlighting differences in the volatile organic compound (VOC) profiles. The system achieved 100% classification accuracy between PRE and POST stages and effectively distinguished all flour types. Integrating this e-nose into kitchen equipment offers a concrete opportunity to optimize leavening by identifying the ideal endpoint, improving reproducibility, and reducing waste. In future applications, sensor data could support feedback control systems capable of adjusting fermentation parameters like time and temperature in real time.

## 1. Introduction

In recent decades, technological advancements, particularly in sensors, IoT (Internet of Things), and artificial intelligence (AI), have revolutionized food quality management [1], introducing the concept of “Food Processing 4.0”, which refers to the integration of robotics, smart sensors, AI, IoT, and Big Data in modern food processing. These technologies are key enablers of improved quality control, safety and production efficiency, and sustainability by reducing waste and optimizing resource use [2,3,4,5,6]. The possibilities of implementing IoT sensors in various household and industrial appliances, such as cooking robots and ovens, can enhance control over food processing and preparation times by real-time monitoring of critical parameters like temperature, humidity, volatile compound emission, gas levels, and pH. This surge is largely driven by advancements in nanotechnology and biotechnology, which have sped up the miniaturization of sensors [7,8].

The integration of sensors, IoT, AI, and machine learning has become particularly relevant for the real-time control and optimization of leavening and fermentation in food processing [9,10]. These processes are crucial in bakery foods since they depend on consistency and quality of the products. In baking, the preparation of dough, including both the ingredients and processing conditions, plays a crucial role in determining the macroscopic structure of baked goods, which in turn influences their appearance, texture, taste, and flavor [11,12]. To form this structure, the ingredients are mixed and kneaded, the dough is leavened, and then it is baked. 

Leavening allows the dough to rise and become airy, contributing to light and airy baked goods and a tender crumb desirable in bread production [13]. This process is driven primarily by the activity of yeast or other leavening agents, such as sourdough starter cultures, which introduce microorganisms into the dough [14]. During leavening begins the fermentation process that is responsible for more than just the volume of the dough, it also shapes its flavor, texture, and aroma, making it a central aspect of high-quality baking production [15]. In particular, volatile organic compounds (VOCs) play a key role in the flavor and aroma determination. They take shape during the leavening process, are increased during fermentation and are enhanced upon baking. Taste and aroma of baked goods are two of the most powerful factors influencing a consumer’s decision [16].

During fermentation, yeast, which is typically used in leavened goods, consumes the sugars present in the flour and produces carbon dioxide and alcohol as byproducts [17]. The carbon dioxide gas causes the dough to expand, creating the airy structure that is so prized in bread. At the same time, the alcohol evaporates during baking, leaving behind a range of VOCs that, since they contribute to the distinctive aromas, flavors, and taste [18], are crucial to the overall sensory experience of baked products. These compounds, which include aldehydes, alcohols, esters, and organic acids, are responsible for the complex and sometimes subtle fragrances that characterize freshly baked bread [19]. The formation of VOCs during the fermentation phase is due to microorganisms that break down sugars and starches into simpler compounds [20]. This is the stage at which the flavors of the dough begin to develop. However, the diversity and intensity of volatile organic compound (VOC) production are heavily influenced by several factors, including the type of leavening agent used [21,22], humidity, the duration and temperature of the fermentation process, and the characteristics of the flour [23]. Low temperatures can slow down the yeast fermentation process, reducing the rate of gas production and potentially requiring more time for the dough to ferment. Conversely, increasing the temperature may lower relative humidity, accelerate the fermentation rate, and shorten the overall duration. Temperature affects the aromas produced by yeast [24], while humidity influences both temperature and fermentation time. This timing is crucial; short proofing leads to dense, low-volume bread, whereas longer, cooler fermentation lowers pH and increases volume, enhancing flavor through colloidal changes in the dough [25,26,27].

Flour strength is a key factor influencing fermentation and leavening, as it affects gluten formation, elasticity, and extensibility [28], and consequently the profile of volatile compounds released during these processes. In practice, the higher the protein content, the stronger the flour, as it enhances its ability to form gluten. Specifically, the proteins glutenin and gliadin, when they come into contact with water (or a liquid that contains water, such as milk), combine to form gluten. This gluten network provides structure and elasticity to the dough. Gliadin gives the dough extensibility, allowing it to stretch, while glutenin provides elasticity, making the dough resilient and able to return to its original shape, like a rubber band. Additionally, the flour classification system used in Italy also includes the W and P/L value to define flour strength. They are determined through a mechanical stress test using a Chopin Alveograph machine. The W value measures flour strength and its resistance to deformation, which correlates with its elasticity. The P/L ratio describes the balance between the toughness and extensibility of the dough. Together, these values offer an accurate description of the dough’s performance under real-world conditions and its suitability for specific baking applications.

Traditional methods of monitoring dough leavening, such as manual inspection, rely on sensory cues like touch, sight, and smell to assess the dough’s progress and are often inadequate, since continuous invasive measurements of dough may cause its collapse. Additionally, these methods can be subjective and imprecise, leading to inconsistencies in product quality. Decisions regarding yeast concentrations and fermentation durations often rely on the baker’s experience rather than scientific research, which can lead to over- or under-fermentation, adversely affecting quality [29]. These challenges highlight the need for more objective and accurate tools to ensure consistent results during dough preparation. 

Today, MOS sensors, originally developed in the second half of the last century, are widely used in food quality, safety, and traceability, thanks to their integration with advanced information technologies and their inherent sensitivity (precision) and speed. Many studies have reported the effective use of sensor array systems, also referred to as e-noses, combined with machine learning techniques for different applications and processes, such as to characterize food safety and geographical origin [30,31,32]. In the literature, e-nose applications have been reported to monitor the volatile organic compounds (VOCs) that are released during the baked product preparation, focusing on fermentation and cooking process [33,34,35,36,37,38]. 

The aim of this study is to explore the use of an S3+ device (designed by Nano Sensor Systems s.r.l.; Reggio Emilia, Italy) equipped with MOS nanosensors (e-nose) to monitor dough leavening in real time through the analysis of VOCs. The innovative aspect of this research lies in the integration of a miniaturized sensor array into a planetary mixer, enabling continuous feedback and reducing the need for manual intervention. Using commercial Saccharomyces cerevisiae as the leavening agent, we evaluated the impact of different flour types (W200, W250, W390) on VOC profiles during a 1.5 h fermentation, as well as in the pre-leavening (PRE) and post-leavening (POST) phases. 

Additionally, SPME-GC-MS analysis was performed on PRE and POST samples to validate the sensor data and provide a comparative assessment. The findings suggest that sensor systems integrated with IoT and machine learning technologies offer a promising path for process automation, requiring specific expertise mainly during the initial training phase. These systems provide a precise, non-destructive, and real-time method for leavening control, ensuring consistent quality, reducing waste, and supporting both artisanal and industrial baking.

## 2. Materials and Methods

### 2.1. Dough Preparation and Experimental Setup

Three different types of wheat flour, characterized by distinct strength values (W200, W250, and W390), were used for dough preparation. The ingredients were purchased from a local retail store. The recipe for the experimental preparation of the dough is given in Table 1.

Flours with a protein content of 10–12% (10–12 g of protein per 100 g of flour) and W of 220–300 P/L ≤ 0.7 are considered medium-strength flours, suitable for most baking applications. Flours with a protein content higher than 12%, W ≥ 300, and P/L ≤ 1 are considered strong flours, ideal for products that require a robust structure, such as bread and pizza. Flours with a protein content lower than 10%, W160–200, and P/L ≤ 0.6 are considered weak flours, more suitable for cakes and pastries.

Each dough sample was prepared by adding water, dehydrated *Saccharomyces cerevisiae* yeast, and sugar into a Kenwood planetary mixer (Kenwood Ltd., Havant, UK; model KCL95.424SI), followed by an initial mixing step. Subsequently, the corresponding flour type and salt were added, and mixing continued until a homogeneous dough was obtained. The doughs were then left to leaven at room temperature (22 ± 1 °C) for 1.5 h. Although only room temperature was monitored in this study, future improvements would include the integration of humidity and kneading speed sensors, which are also critical for fermentation dynamics.

**Figure 1:**
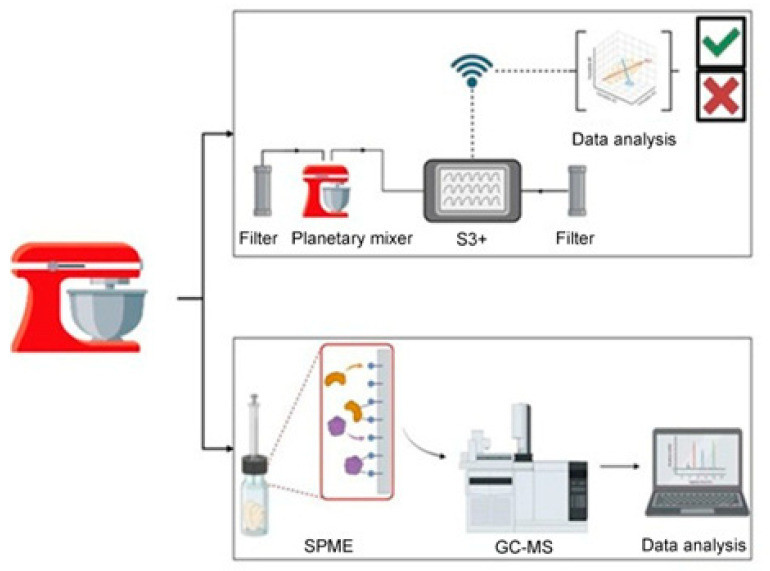
Schematic representation of the experimental setup used to monitor volatile compounds during dough leavening. The upper panel shows the real-time analysis performed with a custom-made electronic nose (e-nose) based on MOS gas sensors, enabling wireless data acquisition and classification of aroma profiles. The lower panel illustrates the complementary solid-phase microextraction followed by gas chromatography–mass spectrometry (SPME-GC-MS) approach.

To analyze the volatile compounds released during the leavening process, two complementary techniques were employed. A custom-designed electronic nose (e-nose) based on metal oxide semiconductor (MOS) gas sensors was used for real-time monitoring of gas emissions, while solid-phase microextraction followed by gas chromatography–mass spectrometry (SPME-GC-MS) provided a detailed characterization of the volatile profile. The combined approach aimed to capture both global aroma trends and specific volatile compounds associated with dough fermentation. The experimental setup for both techniques is illustrated in Figure 1.

### 2.2. S3+ Electronic Nose and Gas Sensor Technology

The S3+ device [39] (designed by Nano Sensor Systems s.r.l.; Reggio Emilia, Italy; www.nasys.it, accesed on 25 November 2024) was equipped with two sets of triple MOS gas sensors designed for volatile organic compound (VOC) detection. Each set included sensors based on SnO_2_ and SnO_2_ doped with Au nanoparticles to enhance selectivity and sensitivity [40,41]. The sensors were placed inside a sealed stainless-steel sampling chamber, where air samples from the headspace of the dough were analyzed.

The core of the S3+ system consists of a sensor array chamber, a fluidic circuit, an electronic control system, and a data processing unit. The sensor chamber houses six metal oxide (MOS) sensors: two pure SnO_2_, two SnO_2_ doped with Pd, and two SnO_2_ doped with Au, all operating at a working temperature of 500 °C (Table 2). The chamber dimensions (11 × 6.5 × 1.3 cm) were optimized to allow controlled airflow through the sensing elements, ensuring consistent exposure to the sample headspace.

### 2.3. Sensor Integration with the Planetary Mixer

**Figure 2:**
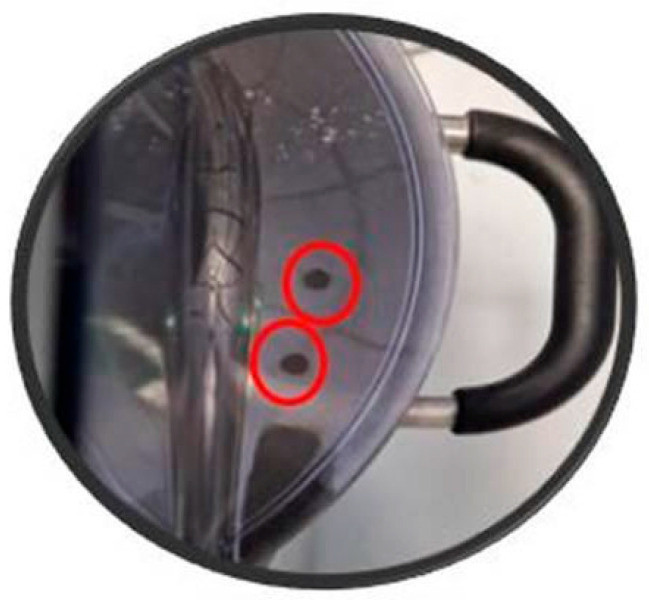
Detail of the upper closure of the planetary mixer showing the perforated section used for gas sampling during VOC monitoring.

The S3+ device was connected to a planetary mixer using a polyurethane tube, which was inserted through a custom-made hole in the mixer’s closure system (as shown in Figure 2). This setup allowed the internal pump of the S3+ device to continuously draw volatile compounds released by the dough during the entire leavening process and transport them into the sensor chamber. The integration of this sampling system aimed to provide a non-invasive and real-time monitoring approach.

This represents a preliminary step toward the direct integration of MOS sensors into kitchen appliances for real-time food monitoring [42,43,44]. Future developments will focus on embedding sensors directly within the planetary mixer structure to improve response time, reduce sampling losses, and further optimize the automation of leavening monitoring.

The fluidic system comprises a diaphragm pump (KNF Neuberger GmbH, Freiburg, Germany, model: NMP05B), an electro-valve (Camozzi Group S.p.A., Brescia, Italy, model: K000-303-K11M), and a metal cylinder filled with activated carbon for environmental air filtration. This setup ensures that only the sample volatiles reach the sensors without contamination from ambient air. The solenoid valve at the inlet regulates airflow at a maximum rate of 250 standard cubic centimeters per minute (sccm), controlling sample delivery and purging phases.

The electronic control system continuously monitors and adjusts sensor temperature, records resistance variations, and transmits real-time data via an IoT-enabled cloud-based platform. A dedicated algorithm processes the collected signals, extracting key features used in subsequent statistical analysis.

### 2.4. Sensor Calibration and Data Acquisition

Prior to analysis, the sensors underwent a multi-step calibration process. Initially, an annealing phase was conducted at 500–800 °C for 1–10 h to stabilize the sensing layers. Following annealing, the sensors were subjected to an aging process under clean air conditions to optimize their baseline resistance.

The validation step included controlled exposure to calibration gas mixtures containing ethanol, acetone, and other fermentation-related volatiles at known concentrations. The sensor responses were recorded and analyzed to establish reference curves. The calibration ensured sensor reproducibility and minimized baseline drift over prolonged usage.

For each measurement, the S3+ device operated in cycles of 13 min: 100 s for sensor stabilization, 200 s for sample exposure, and 500 s for sensor recovery. Each dough type was analyzed in triplicate, with 10 replicate measurements per sample. Data were collected at a sampling rate of 1 Hz, normalizing sensor responses relative to baseline resistance (R/R0) to ensure comparability [45].

#### Signal Preprocessing

Before data interpretation, the raw signals collected by the e-nose were preprocessed to reduce noise and improve comparability across samples. Each sensor’s response was first normalized with respect to its baseline resistance (R0), aligning the initial part of each signal to a common reference point. A Savitzky–Golay filter was then applied to smooth the data and reduce high-frequency noise, ensuring that the trend of the signal remained intact. This preprocessing step is essential, as the raw signal can be affected by various factors, such as temperature fluctuations, humidity, system noise, and residual odors from previous samples.

### 2.5. Chromatography–Mass Spectrometry (GC-MS) Analysis

To validate the MOS sensor responses, volatile compound profiles were analyzed using solid-phase microextraction gas chromatography–mass spectrometry (SPME-GC-MS). Approximately 20 g of dough was transferred into sterile 120 mL glass jars, which had been previously perforated to allow insertion of the SPME fiber into the headspace. Headspace extraction was performed using a DVB/CAR/PDMS fiber for 90 min at 30 °C. Analytes were thermally desorbed and injected into a Shimadzu (Shimadzu Corporation, Kyoto, Japan) GCMS-QP2020 system equipped with a MEGA-5MS column (25 m × 0.25 mm × 0.25 μm). Hydrogen was used as the carrier gas at a flow rate of 2.2 mL/min. The temperature program started at 40 °C (held for 1 min), followed by an increase at 4.5 °C/min to 50 °C, then 6.5 °C/min to 80 °C, and finally 15 °C/min to a final temperature of 180 °C. The total run time was 17 min.

Peak identification was performed using three spectral libraries (Nist11, Nist11b, and FFNSC2) with a similarity threshold of >90% for compound attribution. The results provided a detailed volatile profile of the doughs, enabling a comparative assessment with MOS sensor data.

### 2.6. Data Analysis and Statistical Processing

Multivariate statistical techniques were applied to distinguish between different flour types and leavening phases. Linear Discriminant Analysis (LDA) was performed using the normalized sensor responses as input variables [46]. The LDA model aimed to maximize variance between classes (PRE- and POST-leavening) while minimizing within-class variance.

**Figure 3:**
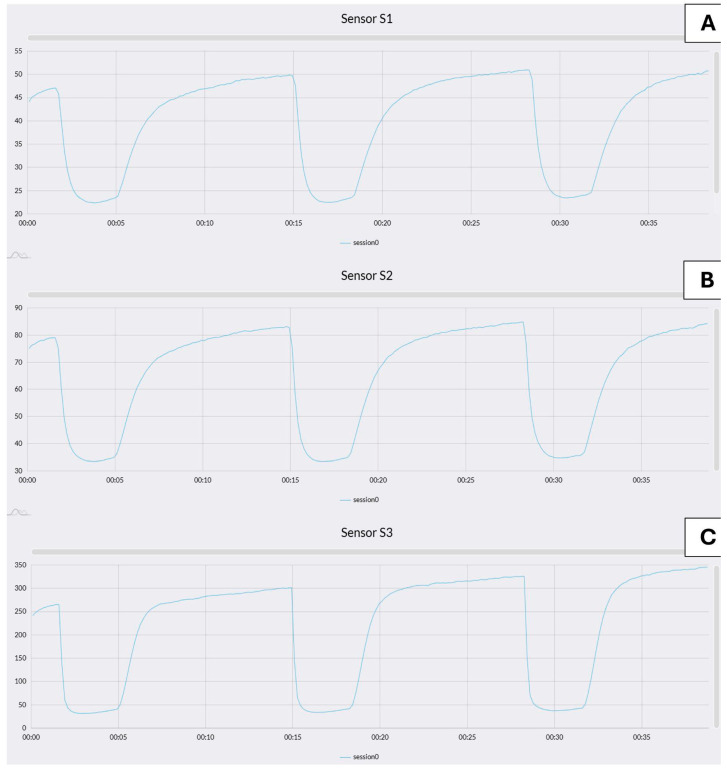
Response curves of the three sensing elements included in the MOS sensor array: (**A**) SnO_2_ (sensor 1), (**B**) SnO_2_ + Pd (sensor 2), and (**C**) SnO_2_ + Au (sensor 3). Each graph shows the electrical resistance (Ω) on the y-axis as a function of time (s) on the x-axis. This curve shows the response to 100 ppm ethanol, a typical fermentation byproduct. The signal profiles reflect the sensor behavior during exposure to clean air followed by ethanol, highlighting the sensitivity and dynamic response of each material.

The sensor response data, expressed as variations in resistance, were transmitted to the cloud and processed using the Microsoft Azure platform, as shown in Figure 3A–C. These plots display the dynamic signal profiles of the three sensing elements composing the sensor array: sensor 1 (SnO_2_), sensor 2 (SnO_2_ + Pd), and sensor 3 (SnO_2_ + Au), respectively. This separation allows for clearer visualization of individual sensor behavior over time. These values were then analyzed through LDA, a technique designed to enhance class separability by emphasizing differences between groups while reducing intra-class variability. Data processing and statistical analysis were carried out using MATLAB R2019b (MathWorks, Natick, MA, USA). To ensure consistency, the raw sensor outputs were normalized relative to the initial acquisition value (R0). For each sensor, the variation between the initial resistance and its minimum value during the measurement period was computed. The ratio R/R0 and the corresponding standard deviation were calculated across all 10 measurement cycles before applying LDA, with an accepted maximum uncertainty of 10%.

Feature extraction (Table 3) from sensor signals included parameters such as minimum derivative, maximum derivative, integral of the response curve, response peak intensity (ΔR), and signal-to-noise ratio. A supervised selection process identified the most relevant features for each sensor, based on a tree-based classifier (random forest). The selected features included mean of the last 60 recorded values, overall mean, minimum response value, signal integral (calculated using Simpson’s Rule), difference between the average of the last five and the first five recorded values, maximum response value, and ΔR (difference between maximum and minimum resistance). These features were used to enhance class discrimination, improving model performance in distinguishing between dough types and leavening phases.

## 3. Results and Discussion

### 3.1. Leavening Process Evaluation Through Volatile Profiles of Different Doughs

Baked goods, with their wide range of products and regional variations, are essential in meeting diverse dietary preferences across the globe [47]. Quality and consumer satisfaction are dependent on taste, smell, and flavor. In turn, they are influenced by the leavening process, which is crucial for achieving consistent baking results. A mixture of more than 500 VOCs, including alcohols, aldehydes, esters, fatty acids, ketones, lactones, phenolics, and sulfur compounds [48,49], influences both taste and aroma. VOCs are present in the initial raw materials and further develop during leavening, as they are produced by various microorganisms (e.g., yeasts, fungi) as metabolites and by-products through catabolic processes, such as glycolysis, proteolysis, and lipolysis [50]. Fermentation is closely linked to the formation of volatiles in fermented foods, although the concentration of these volatiles fluctuates throughout the fermentation process. Thus, dough presents VOC changes between the PRE (unfermented) and POST (fermented) fermentation, which is a crucial developmental stage in the manufacturing process [51]. 

In the literature, studies on volatile compounds in fermented dough samples have shown that the amount of most volatile compounds generally increases as the leavening process progresses [52,53]. An optimal duration of fermentation time enhances gas retention and promotes the expansion of the gluten structure, resulting in improved dough elasticity and texture, allowing the obtainment of high-quality bakery products [54]. We chose a time of 1.5 h as the duration of the leavening phase, and at the end we made the post-leavening analysis. This time is the average duration applied by the kitchen machine for leavening. Cao et al., 2020 [55] in their research reported that all steamed bread samples exhibited increased specific volume and a softer texture when fermented for 60–75 min. 

The leavening process is inherently influenced by environmental factors such as temperature, humidity, and the type and quantity of leavening agents. In our experimental setup, fermentation took place at an ambient temperature (~22 °C), without active regulation of humidity or heating. Despite these uncontrolled conditions, the system proved capable of reliably tracking the dough’s development through VOC analysis. Nevertheless, the integration of the S3+ device into kitchen machines, combined with artificial intelligence models, can be sufficient to assess the leavening state even without active control of temperature and humidity. Machine learning models, when trained on large datasets that account for environmental variability, can effectively distinguish between fluctuations caused by external conditions and genuine changes in the fermentation process. These models are capable of learning how environmental parameters influence the production of volatile compounds, thereby improving predictive robustness and ensuring more reliable assessments under uncontrolled conditions.

Furthermore, as demonstrated in our research, a dynamic approach—based on the temporal evolution of volatile profiles rather than solely on static PRE- and POST-leavening measurements—further enhances the system’s reliability. Continuous analysis of volatile compounds enables real-time monitoring of the dough’s chemical development. Even though absolute values may be affected by temperature and humidity, the shape and rate of change of the volatile compound curves provide more accurate insights into the leavening status, allowing for more precise evaluations that are less sensitive to environmental fluctuations.

This real-time, learning-based approach provides a solid technological foundation for monitoring leavening. However, to fully understand the origin and variability of the VOC signals captured, it is essential to consider the biological and compositional factors that drive fermentation, starting from the yeast activity and flour characteristics. Since yeast metabolizes the sugars in the dough, producing ethanol and carbon dioxide through alcoholic fermentation, it is directly involved in changes in gluten structure and partially in VOC production [56]. A small fraction of the sugars may also undergo secondary fermentation reactions, leading to the formation of aroma compounds [57]. To increase the practical relevance of our study and reduce dough proofing time, we used commercially available dehydrated *Saccharomyces cerevisiae*, in the amount recommended by the recipe based on the quantity of flour used.

The attributes of bakery products depend largely on the type and variety of flour, which is one of the main ingredients in dough preparation [58]. Specifically, chemical, physical, and sensory properties such as texture, volume, and flavor are affected by flour choice, influencing product marketability and recognition by consumers [59]. Previous studies have investigated the impact of wheat flour type, fermentation temperature, and starter origin on acid production and bread characteristics, with findings indicating that the flour type plays the most significant role [60]. Additionally, the variety and type of wheat flour are known to have a substantial impact on aroma development in sourdough bread [61]. In fact, aside from starch, which is the primary component, wheat flour contains several other elements (e.g., gluten, protein content, non-starch polysaccharides, lipids) that influence bread quality. While variations in reducing sugars and protease content may directly affect bread flavor, all other components potentially influence bread aroma through their effects on microbial fermentation [62]. 

#### 3.1.1. VOCs Analysis by MOS-Sensors (e-Nose)

To evaluate the capability of the S3+ device equipped with MOS gas sensors in discriminating dough samples based on their volatile organic compound (VOC) emission during fermentation, Linear Discriminant Analysis was employed. The analysis was performed both continuously over the entire leavening process and in discrete PRE and POST phases. In the continuous monitoring (2D LDA), doughs made from flours of different strength levels (W200, W250, and W390) were compared, while the 3D LDA models were applied to investigate the separation between PRE- and POST-leavening conditions within each flour type. 

**Figure 4:**
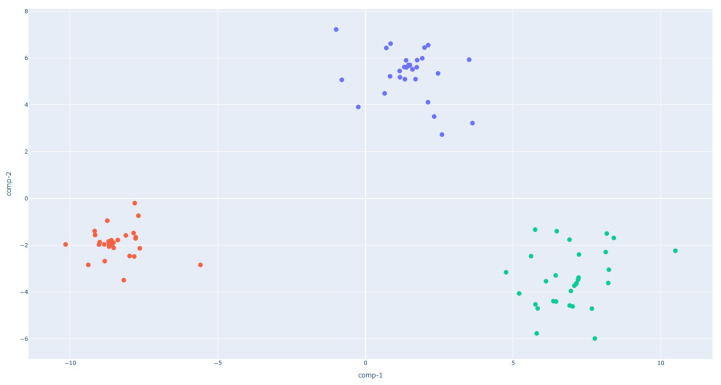
Two-dimensional Linear Discriminant Analysis (LDA) of continuously monitored dough samples during leavening, prepared with W200 (red), W250 (green), and W390 (blue) flours.

The 2D LDA plot for the continuous analysis (Figure 4) demonstrates a clear separation among the three flour types during the leavening process. The clusters corresponding to CONTINUOUS_W200, CONTINUOUS_W250, and CONTINUOUS_W390 are distinctly located in separate regions of the LDA space, confirming the ability of the sensor array to differentiate between doughs of varying rheological properties based on their VOC emission dynamics over time. In particular, the W200 and W250 doughs form tightly clustered and non-overlapping groups, suggesting consistent and distinguishable VOC profiles over time. Notably, the CONTINUOUS_W390 cluster occupies a well-separated area in the LDA space, indicating that the sensor system is sensitive enough to detect differences in fermentation kinetics and metabolite release attributable to the increased gluten strength and higher gas-retention properties of stronger flours. These findings are consistent with previous studies indicating that flour strength significantly influences the release of VOCs during fermentation due to variations in water absorption, gluten network development, and sugar availability [28].

For the 3D LDA analysis of PRE vs POST conditions, the results confirm the discriminative power of the MOS sensor array in identifying leavening progression within each flour type.

**Figure 5:**
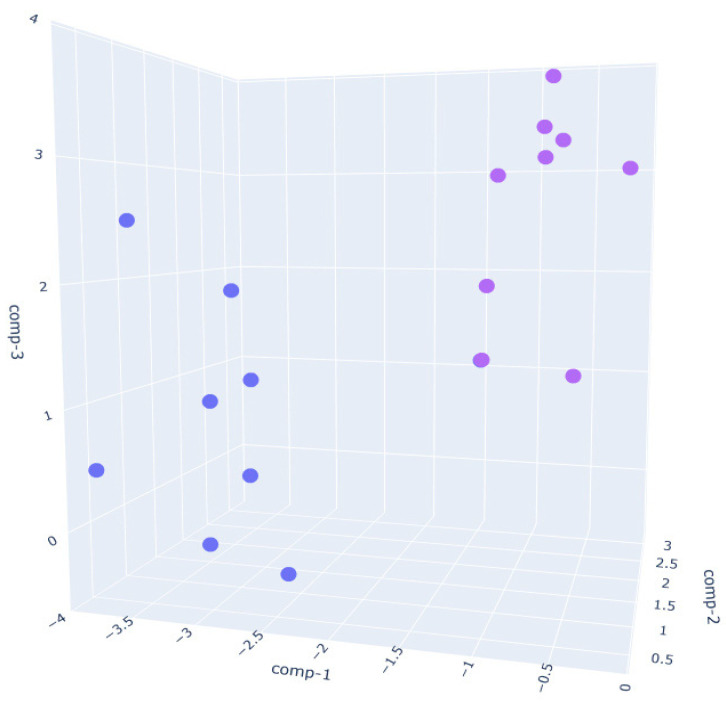
Three-dimensional Linear Discriminant Analysis (LDA) of dough samples prepared with W390 flour in PRE (purple)- and POST (blue)-leavening phases.

**Figure 6:**
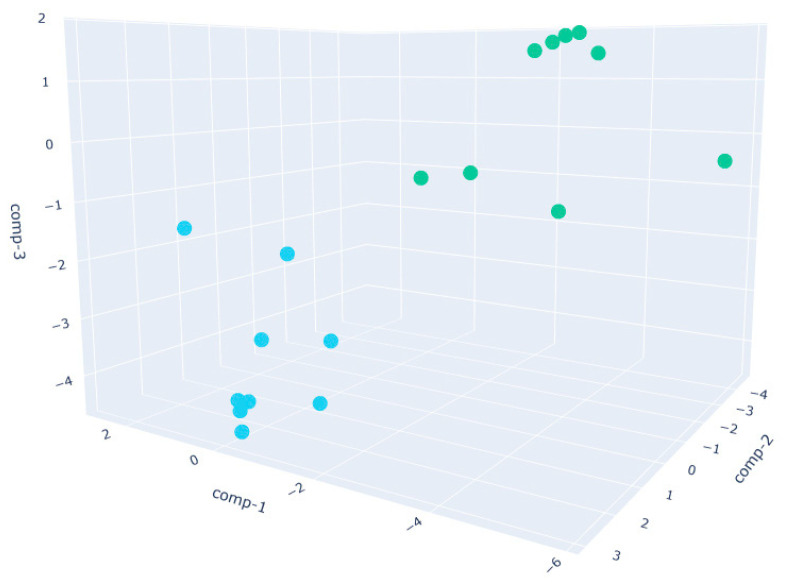
Three-dimensional Linear Discriminant Analysis (LDA) of dough samples prepared with W250 flour in PRE (cyan)- and POST (green)-leavening phases.

**Figure 7:**
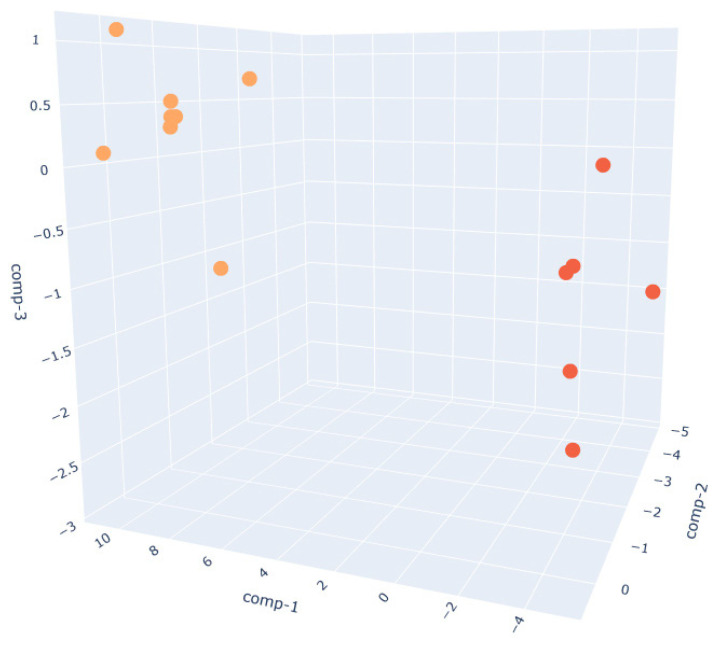
Three-dimensional Linear Discriminant Analysis (LDA) of dough samples prepared with W200 flour in PRE (orange)- and POST (red)-leavening phases. LDA clustering reflects VOC mixture variations; each cluster position correlates specific patterns of VOCs, as confirmed by the GC-MS analysis.

- In the W390 sample (Figure 5), the separation between PRE_W390 and POST_W390 is particularly evident along the first and second discriminant components. The two phases form compact and distinct clusters, indicating significant differences in VOC composition before and after fermentation. This reflects the high gas-retention capacity and slower fermentation kinetics of stronger flours, which lead to more distinct metabolic profiles as fermentation progresses.
- In the W250 sample (Figure 6), the separation is also clear, though slightly more dispersed than in the W390 case. The clusters remain non-overlapping, suggesting that the VOC fingerprint evolves noticeably during the fermentation phase, albeit with more heterogeneity than W390. This aligns with the intermediate gluten strength of W250, which allows for moderate elasticity and extensibility, leading to a well-structured yet less tightly controlled VOC development.
- The W200 sample (Figure 7) exhibits the greatest intra-class variance among the three flour types, as shown by the broader spread of both PRE and POST clusters.This higher signal dispersion observed in W200 samples may be attributed to the intrinsic variability of weaker flours during fermentation. However, sensor-related variability was minimized through baseline normalization, and environmental parameters were kept as constant as possible throughout the trials. Nevertheless, a clear distinction between the two phases is still observed. This broader dispersion may be attributed to the lower gluten content and reduced gas-holding capacity of weaker flours, which can result in less consistent fermentation dynamics and a more variable VOC output. These observations are supported by literature indicating that weaker flours exhibit faster fermentation onset but less stable leavening behavior, with VOC production being highly sensitive to environmental parameters such as humidity and temperature [60].

The clustering observed in the LDA plots reflects differences in the overall composition of the VOCs emitted during fermentation. Although MOS sensors are non-specific, they produce distinct response patterns depending on the concentration and mixture of volatile compounds. These patterns, when processed through LDA, result in the spatial separation of flour types and leavening stages. GC-MS data confirmed that differences in VOC classes—such as alkanes, alcohols, esters, and acids—contribute to this discrimination, reinforcing the robustness of the sensor–LDA system in capturing complex aroma evolution during fermentation.

Overall, the 3D LDA results validate the sensitivity of the MOS sensor-based e-nose system in detecting phase transitions (from PRE- to POST-leavening) in dough fermentation and in discriminating between flour types, not only through their intrinsic chemical profiles but also through the temporal evolution of VOCs. The combination of real-time sensing and multivariate analysis confirms that electronic noses, when properly trained and embedded into kitchen appliances, can serve as robust tools for quality control and automation in food processing. Moreover, the non-invasive nature of the system enables continuous monitoring without altering dough integrity, an essential feature for real-time applications both in domestic and industrial settings.

#### 3.1.2. VOC Detection by SPME-GC-MS

**Figure 8:**
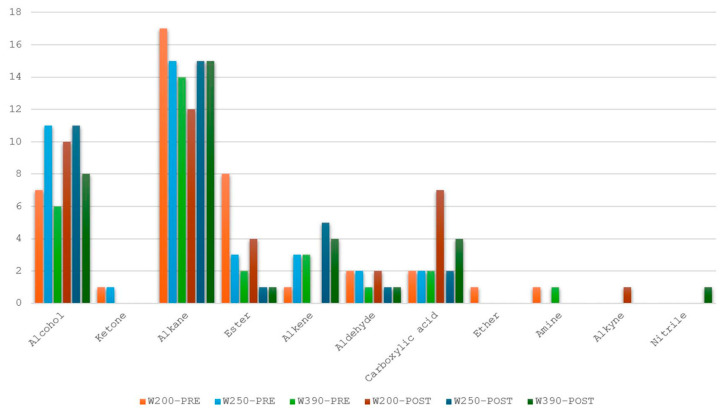
Number of compounds (y-axis) divided into chemical classes (x-axis) present in PRE- and POST-leavening doughs made with flours W200, W250, and W390.

In this study, the conventional SPME-GC-MS method was used for both qualitative and quantitative analysis of the volatile profiles of three types of dough samples, made from flours of different strengths, before and after leavening. The analysis determined the concentration of VOCs and identified the compounds present, with the goal of providing a comparative evaluation against the S3+ device. In our dough samples, obtained from three different flours, a total of 108 different volatile compounds were identified, including alkanes, alcohols, esters, carboxylic acids, alkenes, and aldehydes, in that order of abundance. Ketones, ethers, amines, alkynes, and nitriles were present in trace amounts. Specifically, we found 72 VOCs in pre-leavening and 70 in post-leavening (34 VOCs in common between PRE and POST; 38 compounds specific for PRE, 36 specific for POST). Our findings show a similar total number of VOCs in both PRE- and POST-leavening, but with differences in the types of VOCs between the two phases. Figure 8 illustrates the chemical classes of VOCs detected at the two time points analyzed in the three different doughs. Alkanes, or hydrocarbons, were the most abundant volatiles, showing the highest number compared to other chemical compounds. The number of alkanes was the same in the PRE-leavening samples of W250 and in the POST-leavening samples of both W250 and W390. In contrast, the dough made with W390 showed alkanes only in the PRE-leavening phase, and dough made with W200 flour exhibited a variable number of alkanes in both PRE and POST conditions (14, 17, and 12, respectively). 

**Figure 9:**
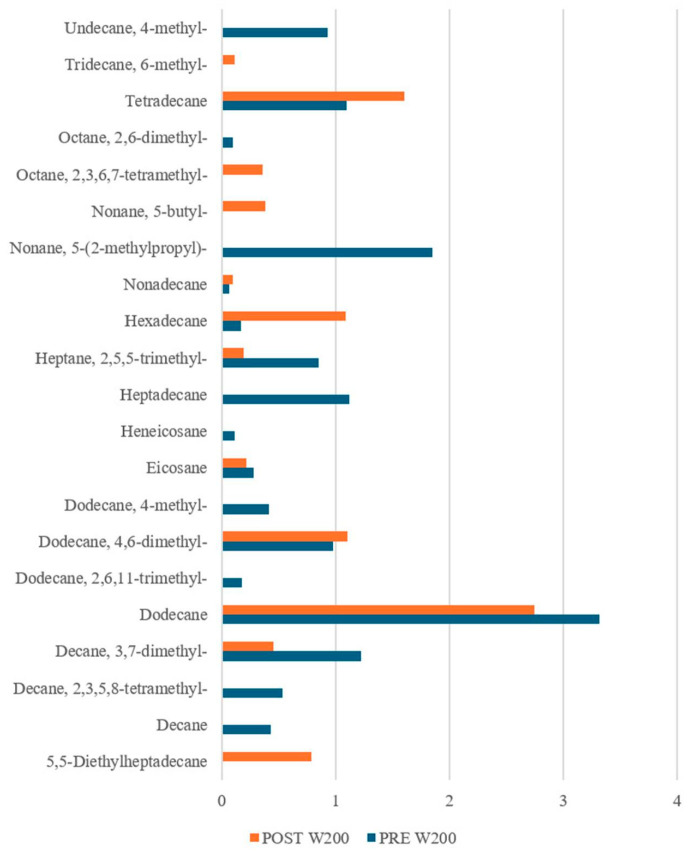
Alkane Compounds in dough samples made with flour of W200, PRE- (blue bars) and POST-leavening (orange bars).

**Figure 10:**
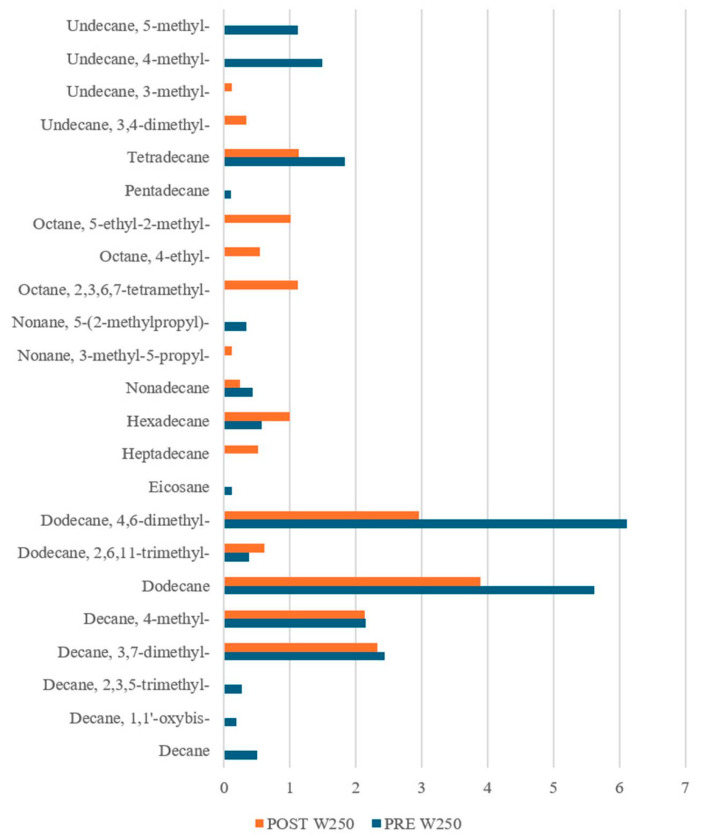
Alkane Compounds in dough samples made with flour of W250, PRE- (blue bars) and POST-leavening (orange bars).

**Figure 11:**
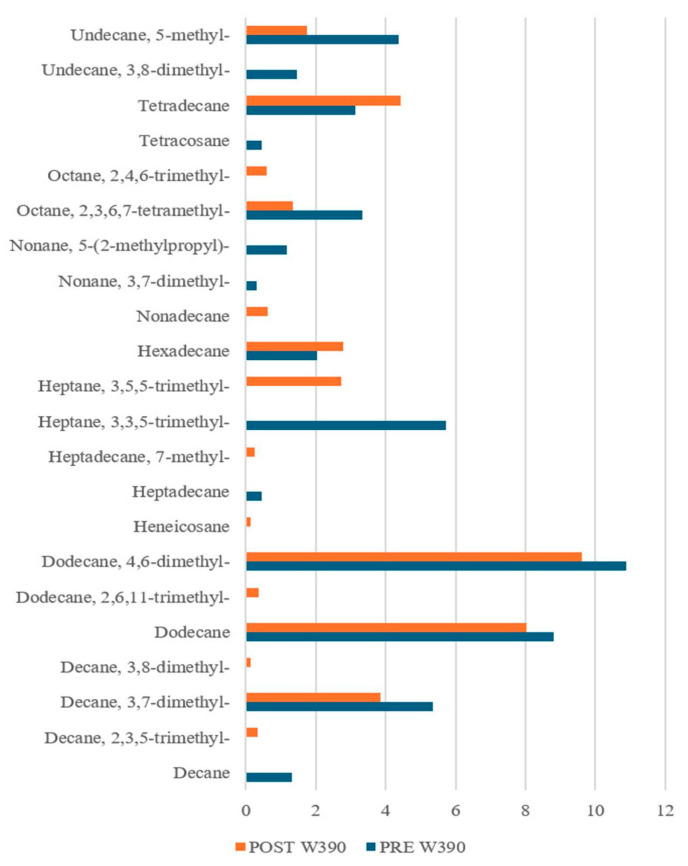
Alkane Compounds in dough samples made with flour of W390, PRE- (blue bars) and POST-leavening (orange bars).

Interestingly, our results (Figure 9, Figure 10 and Figure 11) revealed notable differences not only between the PRE- and POST-leavening phases, but also among the three doughs (W200, W250, and W390). Specifically, several compounds were uniquely present in either the PRE- or POST-leavening phases, as well as in specific dough types. For example, some compounds, such as decane 2,3,5-trimethyl, dodecane 4-methyl, and octane 2,6 dimethyl in W200 and tetracosane in W390, were only present in the PRE-leavening phase. In contrast, in the POST-leavening phase, 5,5-diethylheptadecane, tridecane 6-methyl, and nonane 5-butyl were detected in W200, nonane 3-methyl-5-propyl- in W250, and decane 3,8-dimethyl and heptadecane 7-methyl in W390, among others. Additionally, some compounds, such as decane, were present only in the PRE-leavening phase across all three conditions analyzed. The presence of decane only in the PRE-leavening phase suggests that this volatile compound is a product of initial chemical reactions occurring before active leavening, but it is not continuously produced or detected during the fermentation phase due to changes in the chemical and enzymatic conditions during fermentation, which may not favor its production. The collected GC-MS data highlights the ability to discriminate between doughs based on the fermentation stage (PRE and POST) and the strength of the flour used, supporting the results obtained through e-nose analysis. We identified differences in VOCs not just between the PRE- and POST-leavening stages, but also among doughs prepared with flours of differing strengths. Thus, the strength of the flour likely impacts both the dough structure and the profile of volatile compounds by influencing fermentation dynamics and the production of aroma precursors. Looking at the literature, it is reported that alkanes, saturated hydrocarbons, have been the most abundant chemical class detected in sourdough fermentation followed by esters, alcohols, ketones, aldehydes, and sulfur compounds [63]. Moreover, hydrocarbons are the most common volatile compounds in Tarkhineh, a traditional Kurdish fermented food made from wheat meal (bulgur) and doogh [64,65,66]. In general, hydrocarbons are also the most prevalent volatile compounds in cereals (used as raw material), primarily formed through decarboxylation and carbon-to-carbon cleavage of higher fatty acids. Although these compounds have a high threshold, their contribution to flavor is minimal [67,68]. In the literature, it is also reported that there is no direct correlation between the number of carbon atoms in hydrocarbon chains and the intensity of the aroma, although differences in the overall aromatic characteristics may arise [69]. However, while alkanes are not highly aromatic volatiles, they can still influence the overall aroma profile of the finished product, particularly when combined with other more aromatic volatile compounds, such as aldehydes, esters, or alcohols.

Alcohols, aldehydes, ketones and carboxylic acids are fermentation metabolites that arise through catabolic reactions, such as deamination, decarboxylation, and transamination, which play a crucial role in influencing the sensory characteristics of bakery products [70]. Among the volatile compounds found in our samples, alcohols were the second most abundant chemical class. An increasing trend in the number of alcohols was observed between the PRE- and POST-leavening phases for the W200 and W390 doughs, whereas no change was observed for the W250 doughs. 

During fermentation, the baker’s yeast *Saccharomyces cerevisiae* ferments the sugars and carbohydrates present in the dough, producing primarily carbon dioxide (CO_2_) and ethanol [71]. This process is responsible for the leavening of the dough [72]. Although ethanol is a volatile aroma compound, its sensory impact is less noticeable compared to some of the more flavorful molecules formed as byproducts during fermentation [73]. Nonetheless, ethanol contributes to the dough’s expansion, influencing both the texture and rise of the final product, mainly during baking, since it boils at about 78 °C and partially evaporates. In our samples, at the end of fermentation, the ethanol concentration was higher than at the beginning for all the doughs considered (W200, W250, and W390). On the other hand, compounds such as 1-Hexanol; 2-Isopropyl-5-methyl-1-heptanol and 1-Dodecanol, 3,7,11-trimethyl had a similar or less content in the POST-leavening phase. Our findings may be the result of a combination of factors, including the metabolism of the compounds by the yeast, volatilization during fermentation, oxidation, and binding or entrapment within the dough matrix, in which bubbles were forming, leading to dough rising. According to [74], the different amount of alcohol production may be related to the distinct degradation reaction of amino acids occurring during sourdough fermentation via the Ehrlich pathway, leading to the formation of aldehydes or the corresponding alcohols. Moreover, fermentation temperature also influences the types of volatile compounds generated during the process. At higher fermentation temperatures (35 °C), there is a significant rise in aldehydes and esters, while fewer alcohols are produced compared to lower temperatures (28 °C) [75]. Moreover, similarly to the alkanes, we found some compounds that were exclusive to specific conditions. For instance, 1-Nonanol, 4,8-dimethyl was detected in the PRE-leavening phase of doughs W200 and W250, but not in W390. 1-Nonanol and 2-Decanol appeared in the POST-leavening phase of W200, 1-Undecanol in the POST-leavening phase of W250, and 1-hexadecanol 3,7,11,15-tetramethyl in the POST-leavening phase of W390. These alcohols can contribute a fresh and fruity aroma to the dough, except for the latter, whose presence indicates a fatty and waxy note. They are typically produced during fermentation through yeast activity and may develop into more complex aromas after baking.

As for aldehydes, we observed a low content, which was slightly higher in the PRE-leavening samples than in the POST-leavening samples. This fact can be in line with reactions such as the synthesis of alcohols and acids from aldehydes [76]. Frasse et al. (1993) [77] reported in their study on the influence of fermentation on volatile compounds in French bread dough that aldehydes and alkanes did not increase in *S. cerevisiae* fermented samples. Wang et al. (2020) [51] also reported a low and relatively constant level of aldehydes in both wheat dough samples, whether fermented or not, that were incorporated with buckwheat. Low concentrations of aldehydes are responsible for the fresh and slightly green notes in dough [78]. In addition, the same authors, Wang et al. (2020) [51], found in their study a low and relatively constant level of ketones during fermentation, except for 2,3-butanedione. In our research, ketones were present at a very low level at the PRE-leavening phase only in two samples. The number of acids was low and constant before leavening among the different doughs, while increasing after fermentation, except for the dough obtained from flour W250. In particular, eicosanoic acid had a high content or was detected only in the PRE-leavening phase, while stearic acid levels increased after leavening. Similarly, Tasan (2003) [79] in his research found that the dough fermentation process had no impact on the fatty acid composition of the corn bread. However, he reported that after baking, slight changes were observed in the fatty acid profile; the concentrations of palmitic, stearic, and oleic acids increased, whereas the levels of linoleic, linolenic, arachidic, and eicosenoic acids decreased compared to those in the flour and dough. In addition, Wang et al. (2020) [51] observed that acids and esters became detectable only at the final stages of fermentation, playing a key role in the flavor differences between fermented and non-fermented dough. In our experiment, the number of esters decreased after fermentation. However, among the content of some compounds, such as octadecanoic acid, propyl ester, and sulfurous acid, hexyl octyl ester was found higher at the end of fermentation.

## 4. Conclusions

In recent years, the food industry has embraced “Food Processing 4.0”, integrating robotics, smart sensors, AI, IoT, and Big Data into modern workflows. In this context, the S3+ device equipped with MOS nanosensors has proven highly effective in monitoring dough leavening. It successfully distinguished both fermentation stages (PRE vs. POST) and flour strengths (W200, W250, W390), with LDA analysis confirming clear clustering based on VOC profiles. GC-MS validation supported these findings, identifying over 100 volatile compounds and confirming the system’s ability to detect key differences.

The integration of real-time sensing and multivariate analysis shows that electronic noses, when trained and embedded in appliances, can offer intelligent tools for leavening control and food quality assurance. Looking ahead, devices like S3+ could evolve into closed-loop systems capable of not only monitoring but also managing the process, thanks to AI modules and onboard classification models. This would allow dynamic adjustment of parameters such as time and temperature based on sensor signals, ensuring consistent results, reducing waste, and supporting sustainable and reproducible home and industrial baking.

## Figures and Tables

**Table 1:** Ingredients used for the preparation of dough samples with W200, W250, and W390 flours.

Ingredients	Amount	Unit
Flour	500	g
Water	300	mL
Dry yeast	7	g
Salt	10	g
Sugar	3	g

**Table 2:** Working temperatures of MOS sensors with different dopants.

Type of Sensor	Doping	Working Temperature (°C)
MOX sensor	SnO_2_	500
MOX sensor	SnO_2_ + Pd	500
MOX sensor	SnO_2_ + Au	500

**Table 3:** Overview of the extracted statistical and dynamic features used to characterize the sensor signals. LDA classification models were trained using leave-one-out cross-validation, and 2D/3D scatter plots were generated to visualize the separation between sample groups. These analyses demonstrated the effectiveness of the S3+ device in discriminating between dough types and leavening stages based on VOC profiles. To manage potential outliers, a threshold was established at three times the standard deviation (99.7% confidence level).

Features	Description
Sharpe Forward 25%	Variability index equivalent to the ratio between the mean and the standard deviation, calculated from the beginning of the signal to 25% of it.
Sharpe Back 25%	Variability index equivalent to the ratio between the mean and the standard deviation, calculated from the end of the signal to 25% of it.
Sharpe Forward 50%	Variability index equivalent to the ratio between the mean and the standard deviation, calculated from the beginning of the signal to 50% of it.
Sharpe Back 50%	Variability index equivalent to the ratio between the mean and the standard deviation, calculated from the end of the signal to 50% of it.
Minimum derivative	Calculation of the minimum derivative of the function in the selected interval.
Maximum derivative	Calculation of the maximum derivative of the function in the selected interval.
Integral	Calculation of the integral of the function in the selected interval.
ΔR	Often called excursion range, this feature represents the difference between the maximum and minimum values observed in the time series.
Logarithm of sum	The sum of the natural logarithm of the signal.
Minimum	The minimum value observed in the time series.
Maximum	The maximum value observed in the time series.

## Data Availability

The original contributions presented in this study are included in the article. Further inquiries can be directed to the corresponding authors.

## Abbreviations

The following abbreviations are used in this manuscript:

VOCs	Volatile organic compounds
SPME-GC-MS	Solid-phase micro-extraction coupled with gas chromatography-mass spectometry
MOS	Metal oxide semiconductors
IoT	Internet of Things
